# Daytime physical activity and nighttime glucose levels in individuals with pregnancy hyperglycemia: linking wearable activity trackers to continuous glucose monitoring

**DOI:** 10.3389/fendo.2025.1694758

**Published:** 2025-10-13

**Authors:** Bethany Rand Hallenbeck, Jill M. Maples, Scott E. Crouter, Hollie Raynor, Nikki B. Zite, Kimberly B. Fortner, Samantha F. Ehrlich

**Affiliations:** ^1^ Department of Public Health, University of Tennessee Knoxville, Knoxville, TN, United States; ^2^ Division of Research, Kaiser Permanente Northern California, Pleasanton, CA, United States; ^3^ Department of Obstetrics & Gynecology, Graduate School of Medicine, The University of Tennessee Health Science Center, Knoxville, TN, United States; ^4^ Department of Kinesiology, Recreation, and Sports Studies, University of Tennessee Knoxville, Knoxville, TN, United States; ^5^ Department of Nutrition, University of Tennessee Knoxville, Knoxville, TN, United States; ^6^ Center for Women and Infants, The University of Tennessee Medical Center, Knoxville, TN, United States

**Keywords:** pregnancy hyperglycemia, physical activity, sedentary behavior, precision health, continuous glucose monitoring, digital health, wearable devices

## Abstract

**Introduction:**

Continuous glucose monitoring (CGM) offers a unique opportunity to assess Q6 glucose patterns across the 24-hour day, including nighttime. In individuals with pregnancy hyperglycemia, evidence suggests that optimizing nocturnal glucose levels reduces the risk of large-for-gestational-age births and future metabolic dysfunction. However, the behavioral correlates of nocturnal glucose levels remain poorly understood. Continuous movement devices assess physical activity (PA) and sedentary behavior (SED) across 24-hour days, and to the best of our knowledge, have not been paired with CGM data in individuals with pregnancy hyperglycemia. This secondary analysis of a feasibility trial explored the association of day-time PA and SED with nighttime (i.e., 12–6 AM) interstitial glucose levels in individuals with gestational diabetes mellitus (GDM) or gestational glucose intolerance (GGI).

**Methods:**

Participants (N = 13; ~31 weeks gestation) wore a Dexcom G6 CGM and ActiGraph Insight Watch for 7 days. Mixed effects models examined associations between daytime moderate-tovigorous physical activity (MVPA), light physical activity (LPA), and sedentary behavior (SED) with nocturnal glucose metrics, including mean glucose, time in range (TIR; 60–99 mg/dL), and area under the curve (AUC).

**Results:**

Adjusted models revealed that each 10-minute increase in MVPA was associated with 0.86 mg/dL [95% confidence interval (CI) 0.002, 1.73] higher mean glucose and 313 mg/ dL*min (CI 0.98, 624.55) higher AUC. No associations were observed for total activity, LPA, or SED.

**Discussion:**

These findings illustrate the feasibility and potential of combining CGM with activity monitor data, and the need to further integrate dietary intake data. Improvements in glucose and activity monitoring technology hold great promise for improving scientific and clinical understanding and supporting the development of personalized, data-driven glucose management tools during pregnancy.

## Introduction

Gestational diabetes mellitus (GDM) and gestational glucose intolerance (GGI) are increasingly common complications of pregnancy and are associated with heightened risk of fetal overgrowth, birth complications, and long-term cardiometabolic disease for both mother and child (1–14). Daytime glycemic control has been a focal point in GDM management, partially due to the lack of practical measurement tools for detecting nighttime glucose. However, morning fasting glycemia, which can be seen as a reflection of nighttime glucose, has been associated with large-for-gestational-age (LGA) infants (15). Now with the addition of user-friendly continuous glucose monitors (CGM) to measure nighttime glucose, emerging evidence suggests that nocturnal hyperglycemia (measured between 12–6 AM) is associated with LGA (16) and the need for pharmacologic treatment in pregnancy (17).

Advances in continuous glucose monitoring (CGM) technology have opened new avenues for monitoring glycemia across the 24-hour cycle, allowing researchers and clinicians to identify patterns of dysglycemia that occur overnight. In parallel, wearable activity monitors provide detailed, real-time insights into daily movement behaviors. Together, these technologies offer a powerful, scalable means to assess the dynamic interplay between health behaviors and glucose regulation, with the potential to inform personalized diabetes management during pregnancy.

This secondary analysis of a feasibility trial explored concurrently collected CGM and wrist-worn activity monitor data to investigate associations between daily minutes of moderate-to-vigorous physical activity (MVPA), light physical activity (LPA), and sedentary behavior (SED) with nocturnal glucose metrics in individuals with GDM or GGI.

## Methods

### Data

The present study is a secondary analysis of data collected from a subsample of participants enrolled in a randomized controlled feasibility trial of a behavioral PA intervention for GGI or GDM (ClinicalTrials.gov Identifier NCT04209348). Individuals between the ages of 18–40 with GGI had a 1-hr plasma glucose ≥130 mg/dL on the 50-g glucose challenge test (GCT), measured at 24+ weeks' gestation [i.e., followed by an oral glucose tolerance test (OGTT) that did not meet GDM criteria] (18). GDM could be diagnosed by either the one-step procedure (a 75-g OGTT with plasma glucose: ≥ 92 mg/dL for fasting, ≥180 mg/dL 1-hr post-glucose load, or ≥153 mg/dL 2-hr post-glucose load) or the two-step strategy (a plasma glucose ≥130 mg/dL on a GCT followed by a 100-g, 3-hr OGTT with two or more plasma glucose measurements: ≥95 mg/dL for fasting, ≥180 mg/dL 1-hr post-glucose load, ≥155 mg/dL 2-hr post-glucose load, ≥140 mg/dL 3-hr post-glucose load). Participants were excluded from participating if they were diagnosed with GDM before 24 weeks gestation, were taking glucose lowering medications (e.g., insulin, metformin, glyburide, etc.), or had extenuating pregnancy complications precluding them from PA (determined an obstetrician). Twenty patients were enrolled from the University of Tennessee Medical Center Knoxville; twelve were classified as GGI and eight were classified as GDM. Because of COVID-19 protections, participants opted in to a CGM assessment. The subset of participants who opted-in wore a Dexcom G6 CGM (Dexcom, Inc, San Diego, CA), carried a masked receiver, which was linked to the CGM sensor worn on the posterior upper arm. In order for the CGM sensor to transmit glucose data to the receiver, it must be within 20 feet of the sensor. Participants were given belt clips to help them keep their receiver close. Participants concurrently wore an activity monitoring device, the ActiGraph CentrePoint Insight Watch (hereafter, Insight Watch), on the dominant wrist for seven days, 24-hours each day.

### Assessment of physical activity and sedentary behavior

Insight Watch data was imported into ActiLife software and converted into 1-second epoch and raw CSV files for each participant. Epoch files were run through the Choi et al. wear time algorithm (19) for wrist worn activity monitors in RStudio version 2022.02.03 + 492 statistical software. Raw files were run through the dominant wrist-specific TwoRegressions algorithm (20), which estimated min by min metabolic equivalents of task (METs) for periods in which the devices were worn. Subsequent output RDA files (for each participant epoch and raw file) were merged, and summary results were generated, which included valid wear mins, and mins per day at ≥ 3.0 METs indicative of MVPA, 1.6-2.9 METs indicative of LPA, mins per day ≥ 1.6 METs indicative of total PA, and ≤ 1.5 METs indicative of SED. Days with less than 600 minutes of valid device (i.e., Insight Watch) wear time were then excluded from the analysis.

### Assessment of nocturnal glucose

Glucose was measured using the Dexcom G6 Pro CGM system, which includes a small, wearable sensor and transmitter that records interstitial glucose values every 5 minutes. Participants and their providers were masked to the CGM data (i.e., blind mode) to prevent values from influencing regular behavior. Data from the nocturnal period, defined as 12AM - 6AM (21), were isolated and the mean nocturnal glucose, the percent of time in range (%TIR; 60–99 mg/dL (22)), and area under the curve (AUC) calculated. AUC was calculated in RStudio, using 12AM glucose as the starting point and the trapezoid method (23). All other glucose calculations were performed in SAS Enterprise Guide 8.1 (24).

### Covariates

Gestational age (GA) information was obtained from the electronic medical records (EMR) at UTMCK. GA in days was calculated for each day a participant wore the devices. Maternal age and pre-pregnancy BMI were also abstracted from the EMR at the time of eligibility screening. Data on race and ethnicity, education level, marital status, employment, and number of prior pregnancies were collected via survey administered at baseline.

### Statistical analysis

Medians and interquartile ranges (IQR) were calculated for continuous variables, and frequencies and percentages for categorical variables (20). Mixed effects regression models were created using PROC MIXED in in SAS Enterprise Guide 8.1, with an autoregressive covariance structure, to estimate the association of 10 min blocks of MVPA, LPA, and total activity with nocturnal mean glucose, TIR, and AUC. Variables in the adjusted models were selected *a priori* based on previous literature and further model fitting was performed using backward selection (25), and comparison of AIC, AICC, and BIC scores between models. Initial variables selected *a priori* included pre-pregnancy BMI, GA, GDM vs GGI, maternal age, and number of valid device wear minutes. There was a statistically significant increase in mean nocturnal glucose and AUC by gestational day in participants (P < 0.05) and minutes of valid device wear time also varied between days and participants at baseline. Thus, the final model was adjusted for GA in days and device wear minutes. Pre-pregnancy BMI, GDM vs GGI, and maternal age were not included in the models due to limited degrees of freedom and little impact on the results in statistical models.

We provided preliminary mean differences and confidence intervals, acknowledging the limited sample size from this feasibility trial. Additionally, we provided feasibility results: adherence to the device wear protocol measured by the percentage of wear-time out of total potential wear time and any technical or practical issues that occurred during the device-wear periods.

## Results

Of the 20 participants enrolled in the study, 14 opted to wear the Dexcom CGM device. Of those 14 participants, one participant had an Insight Watch device failure. Of the 13 participants (4 GDM, 11 GGI) with valid data from both devices, there were 90 valid days out of the 91 days of potential concurrent device observation time (26–33 weeks gestation).

Descriptive characteristics of participants are presented in **Table 1**. Most participants identified as White/Non-Hispanic (77%), completed a postgraduate degree (61.5%), and were employed full time (61.5%); all were married or in a civil union. Participants engaged in a median of 61 minutes of MVPA (IQR: 34.0, 95.0) and 455.5 SED minutes (IQR: 377, 543). Median values for mean nocturnal glucose and TIR were 91 (IQR: 81.0, 97.4) mg/dL and 85% (IQR: 58.3, 100.0), respectively. Out-of-range values were rarely below range (i.e., < 1% of values), and primarily reflect values that fall above range (i.e., above 99 mg/dL).

**Table 1:** Characteristics of participants (N = 13).

Continuous Participant Characteristics	*Median (interquartile range)*
Gestational Age in Weeks	30.6 (30.1, 31.6)
Pre-pregnancy Body Mass Index	24.4 (23.4, 29.5)
Age in Years	36.0 (30.9, 37.8)
Minutes of Daily MVPA	61.0 (34.0, 95.0)
Minutes of Daily LPA	342 (252, 400)
Minutes of Daily SED	455.5 (377, 543)
Minutes of Wear Time	884 (757, 955)
Mean Nocturnal Glucose (mg/dL)	90.8 (81.0, 97.4)
Percent Nocturnal Time in Glucose Range (%)	84.7 (58.3, 100.0)
Nocturnal AUC (mg/dL * min)	32,268.3 (28,740.0, 34,582.4)
Categorical Participant Characteristics	*n, %*
GDM	4, 30.8%
White/Non-Hispanic	10, 77.0%
Postgraduate Degree	8, 61.5%
Employed Full Time	8, 61.5%
Married/Civil Union	13, 100%
Number of Previous Pregnancies Resulting in Live Birth	
0	6, 46.2%
1	3, 23.1%
2+	4, 30.6%

MVPA, moderate to vigorous physical activity, LPA, light physical activity, SED, sedentary behavior, GDM, gestational diabetes.

**Table 2** presents the unadjusted and adjusted results of the mixed effects models of the association of PA and SED with nocturnal glucose measures. MVPA was found to be positively associated with mean glucose and AUC (P < 0.05) in the unadjusted model. For every additional 10-minutes of daily MVPA there was a 1.1 mg/dL higher mean nocturnal glucose and a 317.1mg/dL*min higher nocturnal AUC. In addition, for every additional 10-minutes of total PA, there was a 0.28 mg/dL higher mean nocturnal glucose (P = 0.016).

**Table 2:** Estimated associations of 10-minute increments of moderate to vigorous physical activity (MVPA), light physical activity (LPA), total PA, and sedentary behavior (SED) with nocturnal glucose (12AM – 6AM) in individuals with gestational diabetes or gestational glucose intolerance (N = 13).

Model statistics	Independent variable	Mean glucose (mg/dL)	TIR (%)	AUC (mg/dL*min)
Unadjusted Mean Diff (CI)	**MVPA (units of 10 mins)**	1.096 (0.29, 1.90)*	-2.05 (-4.42, 0.3149)	317.15 (25.41, 608.9)*
Adjusted^a^ Mean Diff (CI)	**MVPA (units of 10 mins)**	0.86 (0.002, 1.73)*	-3.38 (-318.36, 311.59)	312.77 (0.98, 624.55)*
	Insight Watch Wear Mins	0.001 (-0.014, 0.018)	0.05 (-87.94, 88.04)	-2.31 (-21.45, 16.83)
	Gestational Day	1.69 (0.59, 2.79)*	-0.06 (-20,319, 20,319)	507.35 (-2689.97, 3704.68)
Unadjusted Mean Diff (CI)	**LPA (units of 10 mins)**	0.27 (-0.002, 0.54)	0.23 (-0.54, 1.00)	63.34 (-30.14, 156.81)
Adjusted^a^ Mean Diff (CI)	**LPA (units of 10 mins)**	0.111 (-0.23, 0.45)	-0.10 (-1.17, 0.96)	46.97 (-74.16, 168.1)
	Insight Watch Wear Mins	0.004 (-0.014, 0.024)	0.03 (-0.03, 0.09)	–
	Gestational Day	1.67 (0.54, 2.81)*	-0.05 (-3.51, 3.41)	–
Unadjusted Mean Diff (CI)	**Total PA (units of 10 mins)**	0.28 (0.05, 0.51)*	–	69.77 (-10.41, 149.95)
Adjusted^a^ Mean Diff (CI)	**Total PA (units of 10 mins)**	0.19 (-0.11, 0.49)	–	71.42(-22.84, 165.68)
	Insight Watch Wear Mins	0.0002 (-0.02, 0.02)	–	3.11 (-13.34, 7.11)
	Gestational Day	1.67 (0.55, 2.79)*	–	–
Unadjusted Mean Diff (CI)	**SED (units of 10 mins)**	0.099 (-0.11, 0.31)	0.46 (-0.13, 1.05)	3.62 (-70.73, 77.98)
Adjusted^a^ Mean Diff (CI)	**SED (units of 10 mins)**	-0.20 (-0.50, 0.11)	0.50 (-0.45, 1.45)	-74.96 (-203.42, 53.49)
	Insight Watch Wear Mins	0.02 (-0.003, 0.04)	-0.004 (-0.07, 0.06)	4.18 (-18.91, 27.28)
	Gestational Day	1.67 (0.55, 2.79)*	-0.04 (-3.43, 3.36)	496.35 (-4273.47, 5266.17)

^a^Adjusted for Insight Watch wear mins and gestational age in days, and participant; *statistically significant P-Value < 0.05; CI = 95% confidence interval; "-" denotes that model could not compute reliable estimates.

After adjusting for device wear time and gestational age in days, the associations of MVPA with mean glucose (mean diff: 0.86, CI: 0.002, 1.73) and AUC (mean diff: 312.77, CI: 0.98, 624.55) were slightly attenuated but remained statistically significant. Adjusted analyses revealed no associations between LPA, total PA, and SED with nocturnal glucose.

## Discussion

This study uniquely combined CGM and wrist-worn device data to explore behavior–glucose dynamics across the 24-hour day. The richness of continuous, timestamped data enabled temporally precise modeling of daily associations using mixed effects models, an approach not possible with traditional self-monitored blood glucose and self-reported activity (21, 26). This method offers improvements in scientific understanding of how lifestyle behaviors influence glycemic control, which can lead to promising avenues for behavioral phenotyping in pregnancy and tailoring interventions based on individual response patterns.

This study shows that integrating CGM with device based, timestamped movement data in pregnancy is both feasible and informative. Participants adhered to wearing both devices 99% of the time with minimal device failures. Moreover, these technologies are rapidly evolving to meet participant and researcher needs. For example, with the release of the new Dexcom G7 CGM, there is now no receiver that must be carried around, and all data can be directly accessed via smartphone.

In this exploratory study of pregnant individuals with GDM or GGI, we found that greater daily MVPA and perhaps total PA were associated with a higher nocturnal mean glucose and AUC. This finding contrasts with existing evidence in nonpregnant and daytime settings, where MVPA is generally associated with improved glycemia (measured in the daytime) in hyperglycemic populations (26–31), and raises important questions about how PA interacts with physiological, behavioral, and hormonal factors in pregnancy. Notably, in the adjusted models, no associations were observed between nocturnal glucose metrics and light-intensity activity, total PA, or sedentary time.

One potential explanation for elevated nocturnal glucose on days with higher MVPA is compensatory dietary intake, such as increased carbohydrate consumption post-exercise. Though diet was not assessed in this study, several studies demonstrate that meal timing and macronutrient composition can significantly impact glycemia (32–34). Similarly, we also did not have data on whether participants were sleeping during the nocturnal period, and poor sleep negatively impacts glucose metabolism in pregnancy (35–37).

Growing evidence suggests that nocturnal hyperglycemia is an important marker of adverse outcomes in GDM. Law et al. (2019) found that poor nocturnal control was associated with increased risk of LGA (16). Similarly, Márquez-Pardo et al. (2020) reported that patterns of elevated overnight glucose predicted pharmacologic treatment needs, i.e., more severe hyperglycemia (17). Moreover, fasting glucose levels, often reflective of nocturnal glycemia, is associated with LGA (15) and postpartum type 2 diabetes risk (6, 38, 39).

This work also highlights the potential for real-time, technology-enhanced diabetes care in pregnancy. As CGM and wearable devices become more common in clinical and at-home settings, they open the door to adaptive feedback systems that respond to individual glucose patterns. Similar strategies have been employed in nonpregnant populations, where CGM, PA, and dietary data are integrated to provide personalized recommendations (40). Such systems could be adapted for use in pregnancy to enhance self-management and clinical decision-making.

This study has several notable strengths. First, it is one of the few to simultaneously capture continuous glucose and PA data in pregnant individuals with GDM or GGI, offering a high-resolution view of real-world behavioral and physiological patterns. The concurrent use of CGM and wrist-worn activity monitors allowed for day-level temporal alignment, enabling more precise estimates of associations between movement behaviors and nocturnal glycemia than are possible with self-reported measures.

Additionally, by focusing on the nocturnal period - a time previously missed in traditional glucose monitoring approaches - this study contributes new insights into an understudied yet clinically relevant window for glycemic dysregulation and its potential links to adverse outcomes.

Some limitations must be acknowledged. The sample size was small and drawn from a pilot feasibility trial, limiting generalizability and statistical power. Importantly, while PA was captured objectively, we do not know the context that the data was capturing, whether the PA was intentional, occupational, related to domestic duties, etc. Most importantly, we did not collect concurrent data on dietary intake or sleep, both of which may confound or mediate the observed associations with nocturnal glucose. The potential for compensatory eating or disrupted sleep on higher-activity days remains an open question and should be addressed in future studies incorporating 24-hour time-use and dietary intake. Future technological advances in dietary assessment stand to help move the field forward. Finally, the classification of SED relied solely on MET values due to limitations in postural detection with wrist-worn devices, which may have led to some misclassification of low-intensity standing behavior as sedentary time.

Building on these preliminary findings, future research should aim to disentangle the complex behavioral, physiological, and contextual factors that influence nocturnal glycemia during pregnancy. High-priority areas include the integration of timestamped dietary intake and sleep measures with CGM and activity data, enabling a more comprehensive 24-hour behavioral and metabolic profile. For example, data from smartphone based ecological momentary assessment tools that can capture images of meals, and daily participant report can provide timestamped diet and sleep data that can be used to capture diurnal and nocturnal relationships between these lifestyle behaviors and continuous interstitial glucose assessment. Such integration could identify whether dietary compensation, altered sleep quality, or circadian misalignment mediates the relationship between PA and sedentary behavior and nocturnal glucose.

Larger, more diverse cohorts will be essential to determine whether these findings generalize across diabetes subtypes (e.g., GDM vs. GGI), racial and ethnic groups, and varying levels of clinical management (e.g., diet-only vs. pharmacologically treated pregnancies). These studies should also consider longitudinal designs to assess whether day-to-day behavior–glucose relationships predict clinical outcomes, such as insulin need, fetal growth, or postpartum glucose dysregulation.

Finally, this line of research sets the stage for the development of real-time, personalized interventions. For example, adaptive interventions that respond to individual glucose or behavior patterns could provide tailored prompts or recommendations for activity, dietary intake, or rest. These technology-driven approaches may enhance self-management support and clinical decision-making, ultimately improving maternal and fetal outcomes in pregnancies complicated by diabetes.

## Conclusions

This work demonstrates the feasibility of combining real-time behavioral and glycemic data to examine 24-hour glucose regulation in pregnancy and serves as a proof-of-concept for the integration of wearable technology into precision prenatal care and highlights opportunities for real-world behavioral phenotyping in the management of diabetes during pregnancy. To the best of our knowledge, we are the first to explore associations between daily PA and nocturnal glycemia using concurrent continuous glucose monitoring and wearable activity tracking in individuals with GDM or GGI. Our findings suggest that greater daily MVPA may be associated with higher overnight glucose levels, a counterintuitive result that may reflect compensatory behaviors or complex physiological interactions during pregnancy.

These results underscore the need for integrated, multi-modal data - including timestamped dietary intake, sleep patterns, and PA - to understand and optimize glycemic trajectories in this population. Future research should build on this foundation to develop personalized, technology-supported interventions that can adapt to individual glucose patterns and behavioral responses, with the goal of improving maternal and fetal outcomes.

## Data Availability

The data analyzed in this study is subject to the following licenses/restrictions: The datasets analyzed during the current study are not publicly available due to the sensitive nature of the data (i.e., pregnancy and diagnostic information on a small sample) that could potentially be linked back to individuals. A summary-level version of the data are available from the corresponding author on reasonable request. Requests to access these datasets should be directed to Samantha Ehrlich, sehrlic1@utk.edu.
